# Comparative analysis of adipose tissue mitophagy and inflammatory markers in obesity and health

**DOI:** 10.1080/21623945.2025.2596407

**Published:** 2025-12-03

**Authors:** Shaghayegh Mohammadzadeh, Mitra Nourbakhsh, Parichehreh Yaghmaei, Atefeh Kashanizadeh

**Affiliations:** a Department of Biology, SR.C., Islamic Azad University, Tehran, Iran; b Metabolic Disorders Research Center, Endocrinology and Metabolism Molecular-Cellular Sciences Institute, Tehran University of Medical Sciences, Tehran, Iran; c Department of Clinical Biochemistry, School of Medicine, Iran University of Medical Sciences, Tehran, Iran; d Department of Surgery, Firoozgar Hospital, Iran University of Medical Sciences, Tehran, Iran

**Keywords:** Obesity, autophagy, inflammation, adipose tissue

## Abstract

Obesity is associated with chronic inflammation and disruptions in cellular homeostasis, including impaired autophagy in adipose tissue. This study aimed to investigate the key mitophagy markers in the adipose tissue of individuals with obesity, compared to healthy controls. A total of 60 participants were enrolled, comprising 30 individuals with obesity and 30 healthy controls. Adipose tissue and peripheral blood samples were collected from all participants. Biochemical analyses included measurement of tumor necrosis factor-alpha (TNF-α), leptin, succinate dehydrogenase (SDH), and oxidative stress markers. Gene expression levels of mitophagy-related genes, PARK2, PINK1, and BNIP3L were assessed using quantitative real-time PCR. Additionally, immunohistochemistry was performed to evaluate BNIP3L protein levels in adipose tissue. Compared to the control group, individuals with obesity showed significantly elevated levels of TNF-α and SDH, along with evidence of oxidative stress. Moreover, the expression of PARK2, PINK1, and BNIP3L was significantly upregulated in the obesity group, suggesting increased mitophagy activity in adipose tissue. These findings indicate heightened inflammation and upregulation of mitophagy pathways in the adipose tissue of individuals with obesity. The upregulation of mitophagy-related genes seems to indicate a possible activation of mitophagy-associated pathways in the altered metabolic and inflammatory environment of obesity.

## Introduction

The prevalence of obesity has increased dramatically in recent decades [], and it is expected to affect over 1.13 billion individuals worldwide by 2030 [1–3]. Obesity increases the risk of serious pathologies, such as cancer, diabetes mellitus type 2, and cardiovascular disorders . This global health crisis is the cause of approximately 1.6 million premature deaths annually from non-communicable diseases such as type 2 diabetes mellitus, cardiovascular disorders, and cancer, and imposes substantial annual healthcare costs [4–6].

Obesity induces profound alterations in cellular metabolism and function. A direct link between autophagy, mitochondrial dysfunction and the progression of obesity has been established by experimental studies on animal models [7,8]. The mitochondrion is the central organelle in processes like energy metabolism, reactive oxygen species (ROS) production, and cellular signalling [9], its homoeostasis depends upon a dynamic equilibrium between its biogenesis and mitophagy (a mechanism for eliminating damaged mitochondria) [10].

Mitophagy is primarily mediated by the pathways through which damaged mitochondria are selectively targeted by proteins like PINK1/Parkin and BNIP3L/NIX for degradation [11,12]. Some metabolic disorders (e.g. obesity, type 2 diabetes, metabolic syndrome, cardiovascular diseases) and neurodegenerative diseases (e.g. Parkinson’s disease, Alzheimer’s disease, amyotrophic lateral sclerosis (ALS), Huntington’s disease, and frontotemporal dementia), are associated with disruption of these processes [13–15].

Recent evidence suggests that mitophagy is activated in response to increased ROS levels. In this process, PINK1 and Parkin play key roles [10]. Furthermore, proteins of the Bcl-2 family are important regulators of mitochondrial stability [16]. Nonetheless, the role of mitophagy markers in human adipose tissue remains poorly understood. ROS-driven activation of inflammatory pathways, such as the NLRP3 inflammasome, is known to exacerbate adipose tissue inflammation. Inflammasome activation and impaired mitophagy may amplify each other, worsening adipose tissue dysfunction in obesity [17].

While the metabolic and inflammatory disturbances associated with obesity are relatively well-characterized, the precise role of mitophagy and its regulators in human adipose tissue, particularly in individuals with obesity, requires further appraisal. In the present study, we investigated the mitophagy markers along with inflammatory indices in the adipose tissue of individuals with obesity, in comparison to healthy ones. Our findings are expected to elucidate the molecular mechanisms underlying obesity and pave the way for new strategies for its prevention and treatment.

## Methods

### Participant selection and grouping

Participants were recruited for this comparative cross-sectional study following a thorough clinical evaluation, and their anthropometric measurements, including height (in cm) and weight (in kg), were recorded. BMI was calculated as kilograms per square metre. Participants with a body mass index (BMI) of > 30 kg/m^2^ were included in the obesity group, while those with a BMI of < 25 kg/m^2^ and no history of extensive weight loss were recruited into the control group. All participants had average or below-average physical activity levels, and no prior history of weight-loss surgery. All subjects had income at or below the population average. Individuals with chronic diseases (such as cancer, cardiovascular disease, or type 2 diabetes), a history of inflammatory or autoimmune disorders (e.g. rheumatoid arthritis, systemic lupus erythematosus), current use of anti-inflammatory medications, pregnancy or lactation, or regular use of tobacco or alcohol were excluded. To minimize selection bias, participants were selected to be as homogenous as possible within groups and not affected by confounders related to inflammation or metabolism.

The study was explained to all participants, and written informed consent was obtained from them.

## Sample collection

Adipose tissue samples were obtained intraoperatively under standardized conditions. In individuals with obesity, omental adipose tissue was collected after at least 12 hours of fasting, during bariatric surgery performed under general anaesthesia. In normal-weight participants, adipose tissue samples were obtained from the omental region during elective laparoscopic cholecystectomy. In all cases, a small section of omental fat was excised using sterile surgical instruments, immediately rinsed in cold saline to remove excess blood, and promptly transferred to the laboratory for molecular and biochemical analyses and immediately stored at −80°C for later analysis. Three millilitres of venous blood were collected from each participant using tubes containing EDTA. Blood samples were centrifuged at 3000×g (10 min) at room temperature, serum was separated, and it was then stored at −20°C for subsequent biochemical analyses.

## Biochemical measurements

Serum levels of leptin (RD191001100 Biovendor, Germany), tumour necrosis factor-alpha (TNF-α) (KPG-HTNF-α Karmania Pars Gene, Iran), and tissue levels of succinate dehydrogenase (SDH) (ZB-13726C-H9648 Zellbio, Germany) were assessed using enzyme-linked immunosorbent assay (ELISA) , according to the manufacturer’s instructions.

For MDA analysis, serum was incubated with thiobarbituric acid (TBA), and absorbance of the resulting colored compound was measured at 535 nm [18]. Total antioxidant capacity (TAC) was determined using a commercial kit (KTAC-96, Kiazist, Iran) using a colorimetric method based on the ability to reduce copper(II) ions., with absorbance read at 490 nm [19].

Measurement bias was reduced by adhering to standardized protocols for sample collection, processing, and biochemical and molecular analyses. All laboratory procedures, were conducted using validated commercial kits and under blinded conditions where laboratory personnel were unaware of participant groupings. The intra-assay and inter-assay coefficient of variation (CV) for all the kits were < 10% and < 12%, respectively. A serum control was analysed in parallel with all study samples to ensure measurement consistency.

## Gene expression analysis

Adipose tissue samples were homogenized and cell lysates were obtained by adding cell lysis reagent (Yektatajhiz, Iran) at a ratio of 1 ml per 50–100 mg of tissue. RNA extraction was performed by silica-based columns (FABRK001, Favorgen, Yektatajhiz, Iran). RNA concentration and purity were assessed using a Nano Drop (Thermo Fisher Scientific, USA).

First-strand cDNA was synthesized using a commercial reverse transcription kit (A101162, Parstous, Iran). The reaction mixture contained reverse transcriptase enzyme (5 μl), 1 μl oligo dT primers (10 µM), RNase-free water (3 μl), and 1 μg template RNA  . The thermal cycling parameters to synthesize cDNA were as follows: 25°C (10 min), 47°C (60 min), and 85°C (5 min), and the synthesis was performed using Bio-Rad thermal cycler (USA). The resulting cDNA was stored at −20°C until further use.

To perform quantitative real-time PCR, SYBR Green Master Mix (70201, Addbio, South Korea) on an ABI StepOne Real-Time PCR System was used (Applied Biosystems, USA). Primers were synthesized by Metabion (Germany) and their sequences are listed in Table 1. The specificity of all primers was verified using Primer-BLAST tool (https://www.ncbi.nlm.nih.gov/tools/primer-blast). The primers showed exclusive specificity for the *PINK1* and *PARK2 (PRKN)* genes, with no off-target amplification detected in the BLAST results. Each reaction mixture included SYBR Green dye (5 μL), forward primer (0.5 μL), reverse primer (0.5 μL), nuclease-free water (3 μL), and 1 μL cDNA template (final volume: 10 µL). The thermal cycling programme includes the following steps in order: initial denaturation (95°C, 5 min), 40 cycles of amplification (95°C for 15 sec, 60°C for 20 sec, 72°C for 30 sec), and a final melting curve analysis.

**Table 1.** Primer sequences for real-time PCR.

Gene	Primer	Sequence (5’- > 3’)	Amplicon length
*GAPDH*	Forward primer	CTTTGGTATCGTGGAAGGAC	126
	Reverse primer	GCAGGGATGATGTTCTGG	
*PARK2*	Forward primer	TGACCAGTTGCGTGTGATTTT	143
	Reverse primer	CGCCTCCAGTTGCATTCATTT	
*PINK1*	Forward primer	TCATCGAGGAAAAACAGGCGG	172
	Reverse primer	CTGCAGCCCTTACCAATGGAC	

The product specificity of the PCR reaction was confirmed experimentally by melt-curve analysis with incremental temperature increases, ensuring the presence of a single, distinct peak for each primer pair. PCR efficiency was evaluated using a five-point serial dilution prepared from pooled cDNA, and the efficiencies for all primer sets were within the acceptable range (90–110%).

Relative gene expression was quantified using the ΔΔCT method, with GAPDH as the reference gene [20]. Standard curves were generated using serial dilutions of pooled cDNA to assess PCR efficiency, which ranged between 90% and 110%. All reactions were run in triplicate.

The selection of primers (Table 1) was based upon PrimerBank and validated via Primer-BLAST software. Working stocks of primers were prepared at 20 µM concentration after resuspension of lyophilized powder according to the manufacturer’s instructions. A 1:5 dilution was made to prepare working solutions for real-time PCR.

## Tissue processing for histology

For histological evaluation, adipose tissue samples were fixed in 10% neutral buffered formalin (Sigma-Aldrich, USA) for 24–72 hours. Dehydration was carried out using ascending concentrations of ethanol (Merck, Germany) (70%, 80%, 90%, and 100%), followed by clearing in xylene (Sigma-Aldrich, USA) and embedding in paraffin wax (Thermo Fisher Scientific, USA). The embedded tissues were sectioned at a thickness of 5 µm using a rotary microtome (Leica Biosystems, Germany) and mounted on silane-coated glass slides (Thermo Fisher Scientific, USA) for subsequent immunofluorescence staining [21].

## Immunofluorescence

Formalin-fixed, paraffin-embedded visceral adipose tissue sections (5 µm thickness) were deparaffinized in xylene and rehydrated through a graded ethanol series (100%, 90%, 80%, and 70%). Antigen retrieval was performed using citrate buffer (pH 6.0) in a microwave oven. Non-specific binding was minimized using 10% goat serum (Thermo Fisher Scientific, USA) in PBS. Sections were incubated overnight at 4°C with primary polyclonal anti-BNIP3L antibody (ab8399, Abcam, UK) at a 1:100 dilution in PBS. After washing, the tissue was incubated with polyclonal Goat anti-Rabbit IgG Alexa Fluor 488-conjugated secondary antibody (A32731, Invitrogen, USA) at a 1:150 dilution for 1.5 hours at 37°C in the dark. Nuclei were counterstained with DAPI (D9542, Sigma-Aldrich, USA). Finally, slides were mounted with glycerol-PBS (Thermo Fisher Scientific, USA) and imaged using a fluorescence microscope (Zeiss, Germany) equipped with the appropriate filter sets: Alexa Fluor 488 was visualized at an excitation wavelength of ~495 nm and emission at ~519 nm (green fluorescence), and DAPI was detected at an excitation wavelength of ~358 nm and emission at ~461 nm (blue fluorescence) [21]. was conducted using Image J Software , .

## Statistical method

Statistical analyses were performed using SPSS software version 26 (IBM Corp., Armonk, NY, USA). Continuous variables, including anthropometric data, serum factors, and gene/protein expression levels, were expressed as mean ± standard error of the mean (SEM). Between-group comparisons were conducted using independent t-tests. The chi-square test was used to compare gender distribution between the two studied groups.

Given the wide age distribution of the participants, we stratified the sample into predefined age subgroups to assess potential age-related differences. Subgroup analyses and a two-way Analysis of Variance (ANOVA) were performed to examine both the main effects of age and obesity status, as well as their interaction.

Spearman’s correlation analysis was performed to evaluate the associations among quantitative variables. A significance level of *p* < 0.05 was adopted for all statistical tests.

## Results

### Anthropometric measurements

Ages range of participants were 24–63 years (mean age 46.28 ± 11.4) and no significant difference was observed between the age and gender of case and control groups. The anthropometric characteristics of the participants are shown in Table 2. The group of individuals with obesity exhibited a significantly greater mean weight than the control. Similarly, mean BMI was significantly elevated in the group of individuals with obesity relative to the control. No significant difference in mean height was observed between the groups.

**Table 2.** Anthropometric characteristics of study participants.

Variable	Control group n = 30	Group of individuals with obesity n = 30	*p*-value
Gender (female/male)	16/14	23/7	0.058
Age (years)	46.50 ± 11.35	45.30 ± 10.7	0.67
Weight (kg)	66.67 ± 2.24	110.73 ± 6.58	< 0.001
Height (cm)	168.48 ± 2.41	169.94 ± 2.34	0.47
BMI (kg/m2)	23.27 ± 0.24	38.04 ± 5.36	< 0.01

BMI: Body mass index.

## Oxidative stress and antioxidant status

The level of malondialdehyde (MDA) was significantly higher in the obesity group compared to the control group, with this increase being statistically significant (Table 3). In contrast, the total antioxidant capacity (TAC) was significantly reduced in individuals with obesity compared to the control group, with this decrease also reaching statistical significance (Table 3).

**Table 3.** Biochemical parameters of study participants.

Variable	Control group n = 30	Group of individuals with obesity n = 30	*p*-value
MDA (µM/mL)	6.84 ± 0.50	15.92 ± 0.72	< 0.001
TAC (nmol of trolox equivalent/mL)	0.854 ± 0.19	0.23 ± 0.12	< 0.001
Leptin (ng/mL)	2.35 ± 0.7	4.92 ± 0.8	< 0.001
SDH (ng/mL)	3.42 ± 0.30	10.52 ± 0.31	< 0.001
TNF-α (pg/mL)	50.55 ± 5.86	198.45 ± 7.80	0.001

MDA: malondialdehyde; TAC: total antioxidant capacity; SDH: succinate dehydrogenase; TNF-α: tumour necrosis factor-alpha.

## Hormonal and inflammatory biomarker analysis

The concentration of the leptin hormone was significantly higher in the obesity group compared to the control group. This marked increase in leptin levels in individuals with obesity was found to be statistically significant. Succinate dehydrogenase (SDH) levels were also significantly elevated in subjects with obesity compared to the control group, demonstrating statistical significance. Additionally, analysis using an independent t-test revealed a significant increase in tumour necrosis factor-alpha (TNF-α) in individuals with obesity compared to the control group, with a highly significant difference (Table 3).

The whole studied subjects were divided into three categories based on their age ranges, including 24–36 years, 37–49 years, and 50–63 years and a two-way ANOVA was performed to examine the effects of age category (three age ranges) on SDH and TNF-α expression levels.

For SDH, the main effect of age range was not significant (F(2,53) = 0.24, *p* = 0.79), and there was no significant interaction between subject groups (obesity and control) and age range categories (F(2,53) = 0.38, *p* = 0.68). Similarly, for TNF-α, there was no significant main effect of age categories (F(2,54) = 0.56, *p* = 0.57), and the interaction between the obesity and control groups and the groups based on the age range was not significant (F(2,54) = 0.69, *p* = 0.50).

Levene’s tests confirmed homogeneity of variances for both variables (all (*p* > 0.05)). Overall, the findings indicate that subject groups (obesity vs. control) significantly influences both SDH and TNF-α levels, whereas age range does not exert a measurable effect nor interact with group status.

## Status of markers of mitophagy

**Figure 1.**
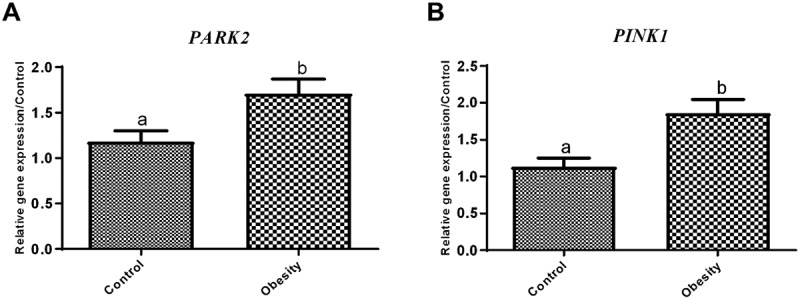
Analysis of data from expression level of genes and protein; **A**. PARK2, and **B**. PINK1. The control group refers to the healthy group, and the obesity refers to the individual with obesity. Data are presented as mean ± SEM; *p* < 0.01, *p* < 0.001.

The expression level of the *PARK2* gene was significantly elevated in individuals with obesity compared to the control group, with a statistically significant difference observed (*p* = 0.01, Figure 1(A)). Similarly, *PINK1* gene expression was notably higher in the group of obesity than in the controls, also demonstrating statistical significance (*p* = 0.01, Figure 1(B)).

Bar charts show relative gene expression levels of PARK2, PINK1 in control and obesity groups. Panel A shows PARK2 expression, with the obesity group displaying a taller bar than the control group. Panel B shows PINK1 expression, also with a taller bar in the obesity group compared with controls. Each bar includes an error bar representing the standard error of the mean.

PINK1 and PARK2 expression levels were also compared in different age categories as mentioned above to investigate if the age range affects the expression of mitophagy markers. The analysis revealed that the main effect of age range was not significant (F(2,54) = 1.830, *p* = 0.170), suggesting that PINK1 expression did not differ significantly across age categories. The interaction between subject groups (control and obesity) and age groups was also non-significant (F(2,54) = 1.957, *p* = 0.151).

Similarly, for *PARK2* the main effect of age range was not significant (F(2,54) = 0.216, *p* = 0.807), nor was the subject groups and age-range categories interaction (F(2,54) = 2.282, *p* = 0.112). Levene’s test confirmed equality of error variances (*p* > 0.05) for both analysis.

We also compared the gene expression levels of *PINK1* and *PARK2* in male and female subjects and found no statistical difference between the two groups (*p* = 0.563 and *p* = 0.549, respectively).

Spearmen’s correlation analysis was performed to explore the associations between mitophagy-related gene expression as the main outcomes and the biochemical parameters (Table 4). PINK1 mRNA expression showed no significant correlation with BMI, age, leptin, MDA, SDH, or TNF-α, but demonstrated a significant inverse correlation with TAC (*r* = −0.372, *p* < 0.01). In contrast, PARK2 expression exhibited broader associations; it correlated positively with BMI, MDA, SDH, and TNF-α, and showed a significant negative correlation with TAC. No significant correlations with age or leptin were observed for either gene.

**Table 4.** Correlation coefficients of PINK1 and PARK2 mRNA with other parameters.

Variable	PINK1	PARK2
BMI	0.109	0.376**
Age (years)	−0.059	0.064
Leptin (ng/mL)	0.185	0.232
MDA (µM/mL)	0.170	0.318*
TAC (nmol of trolox equivalent/mL)	−0.372**	−0.369**
SDH (ng/mL)	−0.085	0.310*
TNF-α (pg/mL)	0.077	0.277*

BMI: Body mass index; MDA: malondialdehyde; TAC: total antioxidant capacity; SDH: succinate dehydrogenase; TNF-α: tumour necrosis factor-alpha. *p < 0.05; **p < 0.01.

## Histological analysis of tissue samples

**Figure 2.**
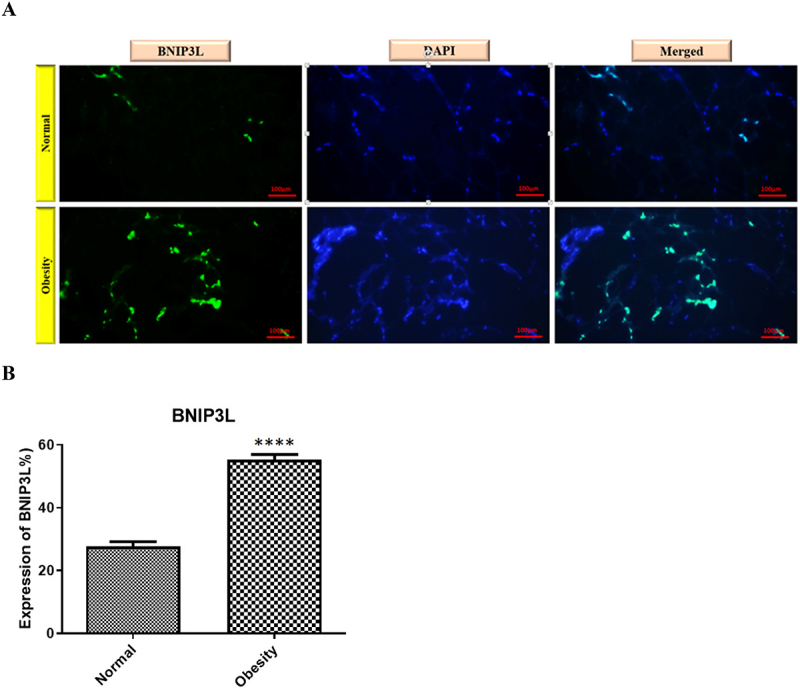
Representative immunofluorescence images showing adipose tissue sections stained with an antibody against the mitophagy-related protein BNIP3L (green, Alexa Fluor) and nuclei counterstained with DAPI (blue). The left column shows the Alexa Fluor signal alone, the middle column shows DAPI staining, and the right column displays merged images. The top row represents samples from normal-weight individuals, and the bottom row represents samples from individuals with obesity. Increased green fluorescence intensity in the obesity group indicates higher expression of the mitophagy marker in omental adipose tissue. **B**. quantification of BNIP3L expression. **** *p* < 0.0001 in comparison with the control group.

As shown in Figure 2, the localization of BNIP3L (indicated in green) in adipocytes from the obese group was identified using immunofluorescence with a specific BNIP3L antibody. Staining of the Nuclear DNA with DAPI (scale bar, 100 micrometres) was used to identify cell nuclei (shown in blue). The results were compared with the healthy control group. A noticeable increase in BNIP3L protein expression was observed in the obesity group compared to the healthy control group. As shown in Figure 2B, the individuals with obesity showed a significant increase in BNIP3L protein levels compared to the control group, which indicates that the statistical results align with the immunofluorescence findings.

Part A shows two rows of immunofluorescence images of adipose tissue, each row containing three panels. In each row, the left panel shows green fluorescence for BNIP3L staining, the middle panel shows blue DAPI nuclear staining, and the right panel shows the merged images. The top row represents normal-weight individuals, with fewer and dimmer green fluorescence areas. The bottom row represents individuals with obesity, showing noticeably brighter and more abundant green fluorescence.

Part B shows a bar chart displaying quantification of BNIP3L expression, with a much taller bar for the obesity group compared with the control group. The bars include error bars representing the standard error of the mean.

## Discussion

Our study demonstrated that the mitophagy-related markers PINK1, PARK2, and BNIP3L were significantly upregulated in the visceral adipose tissue of individuals with obesity. SDH, as a mitochondrial marker, showed elevation in the adipose tissue of subjects with obesity. Additionally, the inflammatory markers (TNF-α and leptin) were elevated in cases with obesity. Serum total antioxidant capacity (TAC) was significantly reduced, while malondialdehyde (MDA) levels were increased, indicating a state of systemic oxidative stress. These findings align with a growing body of evidence linking obesity to mitochondrial dysfunction, oxidative imbalance, and compensatory activation of mitophagy pathways, specifically those involving PINK1/Parkin and BNIP3L, in cellular health and disease states, including obesity [22].

The observed elevation in MDA, a key biomarker of lipid peroxidation, underscores the presence of enhanced oxidative damage in individuals with obesity. This is consistent with previous reports showing that expanded adipose tissue generates excessive reactive oxygen species (ROS) due to mitochondrial dysfunction, NADPH oxidase activation, and infiltration of inflammatory cells [23–25]. Concurrently, the reduction in TAC indicates impaired antioxidant defence mechanisms, which may further exacerbate oxidative stress through diminished scavenging of ROS [26–28]. The interplay between increased ROS production and weakened antioxidant systems creates a vicious cycle, promoting cellular damage, inflammation, and metabolic dysfunction [29].

The upregulation of PINK1, PARK2, and BNIP3L is consistent with previous research, which indicates that the PINK1/Parkin pathway maintains mitochondrial quality control under metabolic stress. For example, according to Fu et al. [7], mitophagy, plays a critical role in controlling mitochondrial quality as well as metabolic homeostasis. Mitophagy is activated in response to increased ROS in obesity, so that it facilitates the removal of damaged mitochondria and protects against further cellular injury [10]. Previous researches showed that in metabolic disorders or oxidative stress, where mitochondrial function and mitophagy are closely intertwined, *PINK1* and *Parkin* act as central mediators, and *BNIP3L* is upregulated [11,12,14]. Our findings reinforce these observations, suggesting that the PINK1/Parkin axis is upregulated as a compensatory mechanism in human obesity and BNIP3L protein expression is increased in adipocytes.

The association between increased mitophagy marker expression and heightened inflammation (as indicated by TNF-α) in our cohort with obesity is in line with the concept that chronic inflammation and oxidative stress are major drivers of mitochondrial turnover in obesity [30–32]. Scherz-Shouval and Elazar [10] and Sidarala et al. [33] both emphasize that ROS and pro-inflammatory cytokines can stimulate autophagic and mitophagic pathways as a protective response to mitigate further cellular damage. To support this adaptive model, our data show concurrent elevation of SDH level and mitophagy genes.

Both PINK1 and PARK2 demonstrated strong negative correlations with TAC, supporting the concept that systemic antioxidant depletion is linked to the activation of mitophagy-related pathways. However, PARK2 showed broader and stronger associations with obesity-related factors, including BMI, lipid peroxidation (MDA), mitochondrial enzyme release (SDH), and inflammatory markers (TNF-α). This pattern implies that PARK2 May be more sensitive to metabolic and inflammatory disturbances than PINK1, possibly reflecting its role downstream of PINK1 in the mitophagy cascade. Collectively, these findings suggest differential regulatory dynamics of the PINK1–PARK2 pathway in obesity, where PINK1 appears consistently upregulated but less variable across individuals, whereas PARK2 expression more closely mirrors the severity of metabolic dysfunction.

A modest upregulation of BNIP3L levels were found during obesity in animal studies [34] This might reflect tissue and species-specific differences in the regulation of mitophagy pathways. Human adipose tissue, with its unique metabolic and inflammatory environment, may require a more robust mitophagic response to cope with chronic metabolic stress and oxidative injury.

The elevated leptin levels in our study confirm the presence of hyperleptinemia, a condition typically associated with obesity, which increases the risk of cardiovascular diseases [35]. Differences in adipose tissue distribution [36], the stage of obesity [37], and methodological variations in leptin measurement [38] can influence such observations. Alternatively, it may indicate leptin resistance, in which circulating leptin does not effectively regulate appetite, energy expenditure, or inflammatory responses despite its elevated levels [39,40].

Activating NF-κB signalling pathway, likely via increased ROS and TNF-α, can upregulate mitophagy receptors, like p62/SQSTM1. Its chronic activation, however, may deplete functional mitochondria, which exacerbates metabolic inefficiency and ROS production. This situation perpetuates tissue inflammation [41–43].

The strong induction of BNIP3L in human adipose tissue highlights species-specific adaptations to metabolic stress. In human obesity, adipose tissue expansion and localized hypoxia may amplify the role of BNIP3L in hypoxia-induced mitophagy [44], driving mitochondrial turnover while inadvertently sustaining inflammatory signalling via DAMPs (e.g. mtDNA) and inflammation [42,44].

The effects of leptin on pro-inflammatory cytokine production (e.g. IL-6, TNF-α) through signalling could exacerbate inflammation [45]. As well, obesity may impair mitochondrial biogenesis via diminished PGC-1α activation [22], compounding the mitophagy upregulative effects. The resulting energy imbalance could drive ectopic lipid deposition and insulin resistance, as seen in hepatic steatosis models [41,44].

The cross-sectional design of our methods prevents us from establishing causality. Relatively small, single-centre subjects limit generalizability to broader populations. The semi-quantitative nature of immunohistochemistry and the lack of post-transcriptional data from real-time PCR as well as lack of data from transmission electron microscopy (TEM) are technical constraints. These limitations should be addressed in future studies to clarify the mechanistic relationships in obesity.

Comprehending the interplay between mitophagy, inflammation, and metabolic health in obesity requires functional studies, such as targeted knockdown of *PINK1*, *PARK2*, or *BNIP3L* in adipocytes, to elucidate their specific roles in regulating mitochondrial quality and inflammatory responses. In parallel, exploring the therapeutic potential of modulating mitophagy pathways, for example, by using pharmacological agents to activate or inhibit BNIP3L, may help determine whether targeting mitophagy can effectively reduce adipose tissue inflammation and improve metabolic outcomes in obesity.

## Conclusion

To summarize, our study demonstrates a significant upregulation of mitophagy-related markers, including PINK1, PARK2, and BNIP3L, in the adipocytes of visceral adipose tissue in individuals with obesity. These molecular changes are accompanied by increased levels of inflammatory markers such as TNF-α, increased oxidative stress (i.e. elevated MDA), and impaired antioxidant defence mechanisms (i.e. reduced TAC). The interplay between mitochondrial dysfunction, oxidative stress, and chronic low-grade inflammation underlies the pathophysiology of the dysfunction. Our results suggest that the activation of mitophagy pathways, particularly through PINK1/Parkin and BNIP3L, may represent a compensatory response to mitigate mitochondrial damage and cellular stress. These findings contribute to elucidating the molecular mechanisms underlying obesity and highlight the potential role of mitophagy modulators as novel therapeutic targets. Future studies should focus on functional analyses of these pathways to restore mitochondrial homoeostasis, reduce oxidative stress, and alleviate inflammation.

## Declaration

The authors declare that they have no conflict of interest.

## Data Availability

Data are available in the following link: https://dataverse.harvard.edu/previewurl.xhtml?token=7041269e-abe1-4805-a4ca-bdd789dba575
